# Some Scientific Aspects of Insanity

**Published:** 1892-01-30

**Authors:** 


### Some Scientific Aspects of Insanity.

### X.—MONOMANIA.

Monomania is a division of mental diseases, comprising a class of patients whose insanity is only partial. Along with this form of insanity is frequently found a greater or less degree of weak-mindedness or dementia. A large proportion of the inmates of asylums are persons labouring under monomania, whether accompanied by dementia or not. A pure and typical case of monomania has one or more fixed delusions, and on most other subjects he is comparatively sane. That is to say, if he is a tradesman he can work at his trade intelligently. If he is an educated man, he can probably read and write and count with advantage, both to himself and others, but there is a failure on the part of his brain to adapt himself to anything new or original in his surroundings. His brain function is not so vigorous or so spontaneous, or so capable of adaptation to change as that of a sane man.

There are three classes of monomaniacs. 1st. Those who have delusions of grandeur, and imagine that they are possessed of extrordinary wealth, or power, or position. They give themselves airs on fitting occasions. This form is generally called "monomania of pride and grandeur." 2nd. Those who have delusions of a depressed nature, who imagine and openly state that people are plotting against them with intent to do them evil. Some of them are so nervous as to resent with suspicion the efforts of their friends to help them. This class is called "monomania of persecution and suspicion." 3rd. Those who imagine that they are being acted upon in a supernatural way, either by their fellow-creatures, or by the denizens of the spirit-world. They, no doubt, are the subjects of disordered sensations and hallucinations of the senses, and being at a loss to account for those sensations or hallucinations, they, in the weakened state of their judging power, refer them to the malice of their enemies, who operate upon their bodies with such mysterious instruments as hidden telephone wires, with poisoned air, or with mesmerism. A favourite expression with many of them is that they are being spiritualised.

It is very rarely that a case of monomania recovers, and they are, therefore, regarded as forming part of the chronic population of our asylums. As the question of their cure is one which, on account of its vagueness, need not be discussed, the question of their care and treatment is the only one remaining to be dealt with.

As any remarks which might be made with regard to the care of monomaniacs would apply equally well to the care of other chronic masses of the insane, it is only necessary to deal with some special features in this form of insanity under the head of care.

As a rule their bodily health is good, they take their food well, and sleep well. Some of them, however, are subject to tubercular disease of the lungs, and it is incumbent upon the attendant to watch carefully any symptoms indicating loss of appetite, falling off in flesh, or cough. When these patients have ordinary colds it is necessary to guard them against draughts, to see that they are properly clad, and generally well cared for. The monomaniacs most liable to consumption are those who have delusions of persecution or suspicion. The great mental symptom is, of course, the presence of a fixed delusion. It should always be borne in mind by the attendant that the delusion is a fixed one, and that no amount of argument or scoffing on the part of others can alter its fixity. It would be as inhuman and ridiculous to ask the lame to walk, and the blind to see, as to expect the monomaniac to think correctly or to judge correctly when ordered to do so. For the same reason that it is considered discourteous to jeer at a man who is physically crippled, it ought always to be remembered, especially by attendants upon the insane, that it is highly reprehensible to make fun of the mental aberrations of these patients.

The delusions of monomaniacs may be divided into two kinds. (1) Those that render the subject actively dangerous to himself and others, and (2) those that are harmless.

The fixed delusions which may render a patient dangerous to himself are those in which he conceives that he has a special divine message ordering him to mutilate himself in one of many ways, or to starve himself, or to commit some highly dangerous feat, such as climbing to the roof of a building or leaping from a high window. The most common delusions which render a patient dangerous to attendants or to his fellow-patients are those in which he imagines that he is being acted upon through them in some supernatural or mysterious manner, or that they are reading his thoughts or conspiring against him. He may have delusions, or hear voices directly commanding him that he is ordained by heaven to execute vengeance upon certain of his fellow-creatures.

The harmless delusions of monomaniacs may lead them into extravagant manner of dress, the assumption of absurd attitudes, and other equally insane but harmless actions, all of which ought gently and firmly to be discountenanced by the officials and attendants in an asylum.

The only advice that remains to be given with regard to the delusions of monomaniacs is that an attendant should always study his patients, make himself intimately acquainted with their most trying whims and mannerisms; then only can he know with certainty which patient's delusions are dangerous and which are harmless.

These monomaniacs are, as a class, very good workers. It does not require much inducement to get them to perform all and every kind of work or to enter into every sort of amusement. The duty of the attendant in this respect is, therefore, a light one, but it is essential for the patients' well-being and happiness that they should be as constantly employed as possible.

